# RyR2 Modulates a Ca^2+^-Activated K^+^ Current in Mouse Cardiac Myocytes

**DOI:** 10.1371/journal.pone.0094905

**Published:** 2014-04-18

**Authors:** Yong-hui Mu, Wen-chao Zhao, Ping Duan, Yun Chen, Wei-da Zhao, Qian Wang, Hui-yin Tu, Qian Zhang

**Affiliations:** 1 Department of Physiology, School of Medicine, Zhengzhou University, Zhengzhou, Henan, China; 2 Department of Pathophysiology, School of Basic Medical Science, Xinxiang Medical College, Xinxiang, Henan, China; 3 Department of Biological Engineering, University of Henan, Kaifeng, Henan, China; 4 Department of Emergency Medicine, University of Nebraska Medical Center, Omaha, Nebraska, United States of America; Sackler Medical School, Tel Aviv University, Israel

## Abstract

In cardiomyocytes, Ca^2+^ entry through voltage-dependent Ca^2+^ channels (VDCCs) binds to and activates RyR2 channels, resulting in subsequent Ca^2+^ release from the sarcoplasmic reticulum (SR) and cardiac contraction. Previous research has documented the molecular coupling of small-conductance Ca^2+^-activated K^+^ channels (SK channels) to VDCCs in mouse cardiac muscle. Little is known regarding the role of RyRs-sensitive Ca^2+^ release in the SK channels in cardiac muscle. In this study, using whole-cell patch clamp techniques, we observed that a Ca^2+^-activated K+ current (*I_K,Ca_*) recorded from isolated adult C57B/L mouse atrial myocytes was significantly decreased by ryanodine, an inhibitor of ryanodine receptor type 2 (RyR2), or by the co-application of ryanodine and thapsigargin, an inhibitor of the sarcoplasmic reticulum calcium ATPase (SERCA) (p<0.05, p<0.01, respectively). The activation of RyR2 by caffeine increased the *I_K,Ca_* in the cardiac cells (p<0.05, p<0.01, respectively). We further analyzed the effect of RyR2 knockdown on *I_K,Ca_* and Ca^2+^ in isolated adult mouse cardiomyocytes using a whole-cell patch clamp technique and confocal imaging. RyR2 knockdown in mouse atrial cells transduced with lentivirus-mediated small hairpin interference RNA (shRNA) exhibited a significant decrease in *I_K,Ca_* (p<0.05) and [Ca^2+^]i fluorescence intensity (p<0.01). An immunoprecipitated complex of SK2 and RyR2 was identified in native cardiac tissue by co-immunoprecipitation assays. Our findings indicate that RyR2-mediated Ca^2+^ release is responsible for the activation and modulation of SK channels in cardiac myocytes.

## Introduction

Small-conductance Ca^2+^-activated K^+^ (SK or K_Ca_2) channels are a subfamily of Ca^2+^-activated K^+^ channels (K_Ca_) observed in neuronal and non-neuronal tissues [1]–[3]. One SK channel, SK2, is expressed in human and mouse cardiac muscle and is highly expressed in the atria compared with the ventricles [4], [5]. The selective knockout of SK2 channels in the mouse revealed multifaceted functions of this channel in cardiac myocytes [6], [7]. SK2 channels are important in the configuration of the action potential in atrial myocytes, especially during the late phase of cardiac action potential repolarization, and in regulation of the heart rhythm and rate under physiological conditions [7], [8].

SK channels are voltage-insensitive and Ca^2+^-dependent. These channels link the intracellular calcium concentration to a wide variety of cellular processes [1], [9]. The calcium sensitivity of SK channels depends on calmodulin, which is constitutively bound to the C-terminal domain of the channel. The binding of calcium to calmodulin results in a conformational change of the channel, which leads to the opening of the channel pore [10], [11]. Intracellular Ca^2+^ ions are derived from the influx of Ca^2+^ into the cell through voltage-dependent Ca^2+^ channels (VDCCs) and by the release of Ca^2+^ from internal Ca^2+^ stores [12].

Ca^2+^ functions as a second messenger and mediates its own release from internal stores through the activation of ryanodine receptors (RyRs) and inositol 1,4,5-trisphosphate receptors (IP_3_R) [13]. RyRs are cation-selective channels that release Ca^2+^ from an intracellular Ca^2+^ storage compartment, the sarcoplasmic/endoplasmic reticulum (SR/ER) [14]. Recent evidence has shown that Ca^2+^ release from intracellular Ca^2+^ stores plays a major role in mammalian Ca^2+^ signaling triads formed by voltage-gated Ca^2+^ channels, RyRs, and SK channels in neurons [15], [16]. In a previous study, we documented the molecular coupling of the SK2 channel with a voltage-gated Ca^2+^ channel in cardiac tissue [17]. Here, we investigate the potential role of the RyR Ca^2+^ release channel in the regulation of the SK2 channel in mouse atrial myocytes using electrophysiology and the lentiviral-mediated delivery of small interference RNA (siRNA) against RyR2 to cardiomyocytes. Our study is the first to exhibit functional modulation of RyR2-mediated Ca^2+^ release on the SK2 channel in cardiac myocytes and describes a new signaling pathway for SK channels by which RyRs modulate Ca^2+^ signaling in the heart.

## Methods

### Single cardiac myocyte isolation

Adult C57B L mice were obtained from the Experimental Animal Center of Henan Province, China (No. SCXK-2010-0001). All of the animal care methods and procedures were approved by the Committee on the Ethics of Animal Experiments of the University of Zhengzhou (No. SYXK-2010-0001). This investigation conformed to the Guide for the Care and Use of Laboratory Animals published by the US National Institute of Health.

Single mouse atrial myocytes were isolated using a previously described enzymatic method [7]. Briefly, adult C57B L mice were anesthetized with sodium pentobarbital (80 mg/kg, intraperitoneally). The lack of hind toe pinching-induced withdrawal reflex, reduced respiratory rate, and lack of reaction to a skin pinch over the incised area were used to monitor the efficiency of the anesthesia. The animals were sacrificed by CO_2_ inhalation. The mice hearts were quickly removed and subjected to enzymatic digestion via Langendorff perfusion. Single atrial cells were isolated and stored in a high-K^+^ solution for 2 h at room temperature before the electrophysiological recordings.

### Electrophysiological recording

The whole-cell configuration of the patch-clamp technique was used. The Ca^2+^-activated K^+^ current (*I*_ K,Ca_) was recorded from freshly isolated atrial myocytes at room temperature using the previously described voltage-clamp protocol [7]. An EPC-10 (HEKA Elektronik, Germany) patch-clamp amplifier was used with Pulse 8.67 software (HEKA Elektronik, Germany). In all the experiments, a series resistance compensation of ≥90% was obtained. The currents were normalized to the cell capacity to obtain the current density (pA/pF).

### Confocal imaging

The calcium imaging experiments were performed as described elsewhere [18], [19]. The intracellular calcium ([Ca^2+^]i) transients were recorded on freshly isolated cardiomyocytes or transduced single myocytes previously loaded with the fluorescent Ca^2+^ dye Fluo-3AM (15 µmol/L, Molecular Probes). The samples were divided into five groups, including the control (caffeine alone), ryanodine+caffeine, ryanodine+thapsigargin+caffeine, Lenti-GFP+caffeine and Lenti-siRyR2+caffeine. The control cells were dispersed in Tyrode's solution (mmol/L: 140 NaCl, 4 KCl, 1.1 MgCl_2_, 10 Hepes, 10 glucose, 1.8 CaCl_2_; pH 7.4 with NaOH), and the other groups were dispersed in Tyrode's solution supplemented with the appropriate inhibitor before performing the experiment. All the cells were stimulated at 1 Hz by field stimulation applied by two parallel platinum electrodes to reach steady state. When an inhibitor was used, the cells were first perfused with the inhibitor alone followed by the addition of 5 mM caffeine in the continuous presence of the inhibitor. The imaging data were recorded 1–2 min following the caffeine applications. All the imaging data were recorded in the line-scanning mode along the long axis of the myocyte excited at 488 nm with a confocal laser scanning microscope (Olympus FV1000, Japan) and analyzed by Olympus Fluoview viewer (Japan). The Ca^2+^ level was reported as the fluorescence (F) over the fluorescence min (F_0_), where F_0_ is the resting or diastolic fluo-3 fluorescence.

### Construction of small interfering RNA

Four optimal 19-mer target sequences (GCCATTCCTACAGTGGTAT, location 864, CGTCCACATACTATTACTC, location 4767, AGGACACCATCAATCTGCT, location 6837 and CACAGCCTATCATCAACAA, location 10404) were selected based on the cDNA sequence of mouse RyR2 (accession number NM-023868.2). Four pairs of oligonucleotides encoding shRNAs and a negative control shRNA were designed and chemically synthesized by Invitrogen. These sequences were subcloned into the HIV-based psiHIV-U6 plasmid (Guangzhou GeneCopoeia, China). Recombinant lentiviral vectors were packaged and amplified in 293T cells using the Lenti-Pac™ HIV Expression Packaging Kit (Clontech) according to the manufacturer's protocol. The supernatant of the cultured 293T cells containing lentiviral particles was collected following transfection for 48 h.

### Mouse neonatal cardiomyocyte culture and infection

Twenty-four hour old neonatal mouse hearts were dissected and subjected to sequential digests with a trypsin/DNase II (Sigma) solution at 37°C. The supernatant was collected at 3–5 min intervals. The dispersed cells were pre-plated for 2 h in DMEM (GIBCO) with 10% fetal bovine serum (FBS, Invitrogen). The unattached myocytes were plated in 35-mm dishes in Dulbecco's modified Eagle's medium (DMEM) with 10% FBS, 1% penicillin-streptomycin (PS, Invitrogen), 0.1 mmol/L bromodeoxyuridine (BrdU, Sigma), and 20 µmol/L arabinosylcytosine (Ara-C, Sigma) at 37°C in an incubator with a mixture of air and 5% CO_2_. Following the fusion of 50% of the cultured neonatal myocytes, the cells were infected with the appropriate recombinant shRNA lentivirus products and maintained at 37°C in a 5% CO_2_ incubator for 48 h. The infection efficiency of the cultured NMCMs was detected by flow cytometry (FCM).

### Flow cytometry

The NMCMs infected with lentiviral vectors were harvested with trypsin and washed with cold PBS. The infected cells were monitored by GFP fluorescence. A PE Annexin V Apoptosis Detection Kit I (BD Pharmingen) was used to identify the apoptotic efficiency of the infected cells. The cells in each sample (2×10^5^) were incubated in Annexin V binding buffer containing PE Annexin V and 7-amino-actinomycin D (7-AAD) at room temperature in the dark for 15 min. The flow cytometry analysis was performed using a FACS Calibur system with FACSDiva software (BD FACSCanto II, USA). The counts are expressed as a percentage of the total number of cells counted.

### Real-time PCR

Total RNA from infected neonatal myocytes was extracted using Trizol Reagent (Invitrogen). The isolated RNA was treated with DNase at 37°C. cDNA was synthesized from the total RNA samples by oligo(dT)-primed reverse transcription using a RevertAid™ First Strand cDNA Synthesis kit (Fermantas). The quantitative real-time polymerase chain reaction (PCR) products were detected using a SYBR Green qPCR Kit (TAKARA) by Bio-Rad MiniOption (USA). The PCR primers used to detect RyR2 and GAPDH were: RyR2 (251 bp), Forward: 5′-GAATTCATCATGGATACTCTACC-3′ Reverse: 5′-GTCATGCACATTATCTTCTGCAT-3′; GAPDH (150 bp), Forward: 5′-TGTGTCCGTCGTGGATCTGA-3′, Reverse: 5′-TTGCTGTTGAAGTCGCAGGAG-3′. The RyR2 and GAPDH primers were designed and synthesized by Shanghai Bioasia Company (China). The relative quantification of RyR2 mRNA was determined by normalization of the threshold cycle (Ct) of these genes to a housekeeping gene, GAPDH.

### Lentiviral transduction of adult mouse cardiac myocytes

Freshly isolated atrial myocytes were suspended in modified Tyrode's solution containing (mmol l^−1^): NaCl 113, KCl 4.7, KH_2_PO_4_ 0.6, Na_2_HPO_4_ 0.6, MgSO_4_ 1.2, NaHCO_3_ 12, KHCO_3_ 10, HEPES 10, taurine 30, 5% FBS and 12.5 µM CaCl_2_ for 10 min at room temperature. After the myocytes were pelleted by gravity, they were resuspended with a stepwise increase in [Ca^2+^]i to 1 mM.

The prepared cardiac myocytes were resuspended in MEM containing 1.2 mM Ca^2+^, 2.5% FBS, and 1% PS (pH 7.35–7.45) at room temperature. The myocytes were collected and plated in a 60-mm laminin-coated dish in MEM containing 2.5% FBS and 1% PS. After 1 h of culture, the medium was switched to FBS-free MEM, and an appropriate titer of gene-carrying lentivirus was added to the culture medium. The cells were maintained at 37°C in a 2% CO_2_ incubator for 36–48 h. A recombinant lentivirus containing GFP alone was used as a control. GFP-positive cells were used for the electrophysiological recordings.

### Western blotting

The protein samples were extracted from cultured adult cardiac myocytes transduced with lentivirus containing Lenti-siRyR2 for 48 h. Thirty-five microgram aliquots of the protein samples were separated by 5% gradient SDS-PAGE for RyR2 and transferred to polyvinylidene difluoride (PVDF) membranes (Bio-Rad). Following transfer, the membrane was blocked and incubated with anti-RyR2 antibody (diluted 1∶600, Affinity BioReagents) at 4°C overnight, respectively. The membrane was incubated with a horseradish peroxidase-conjugated anti-mouse IgG secondary antibody (Thermo). The bands were detected by chemiluminescence (Thermo). Quantification of the signals was performed by densitometry (The Discovery Series Quantity One 1-D Analysis Software Version 4.6.2).

### Co-immunoprecipitation

Reciprocal Co-IP was performed as previously reported [17]. Protein samples from the mice atrial tissues were prepared and incubated with anti-SK2 antibody or anti-RyR2 antibody overnight at 4°C, followed by an additional incubation with 30 µl of protein G sepharose (Santa Cruz) for 6 h at 4°C. The precipitated proteins were analyzed with western blotting (see methods).

### Statistical analysis

The data are expressed as the means±S.E.M. Differences between the groups were evaluated by one-way *ANOVA* followed by paired or unpaired Student's *t* tests, as appropriate. The differences were considered significant when *P*<0.05 and are generally indicated by an asterisk (#).

Further details of the experimental procedures are available in the online supplementary material.

## Results

### Inhibition of RyR2 decreases Ca^2+^-activated K^+^ current in cardiac myocytes

**Figure 1:**
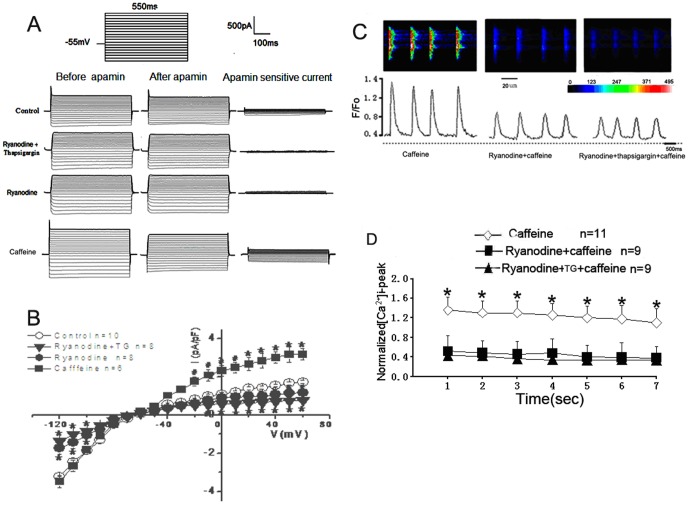
*I_K,Ca_* and intracellular calcium transients recorded from mouse atrial cells. A) Examples of whole-cell *I_K,Ca_* recorded from a mouse atrial myocyte using voltage steps from a holding potential of −55 mV. The voltage protocol used is shown above the current traces. The apamin-sensitive current component was obtained from the subtraction of the total current recorded in the absence and presence of apamin (500 pM), as shown in the right panel. B) Summary of the current density-voltage relationships of the apamin-sensitive current obtained from four groups. The whole-cell current-voltage relationships were obtained by applying 10 mV voltage steps for 500 ms between −120 and +60 mV from a holding potential of −55 mV. A significant difference in the apamin-sensitive current among the four groups was found by *ANOVA*,*P<0.01, #<p<0.05. C) Examples of the line-scan images for single atrial myocytes (top) and the corresponding fluorescence intensity-time curves (bottom). [Ca^2+^]i transient (F/F_0_) induced by 5 mM caffeine was suppressed by 20 µM ryanodine and 2 µM thapsigargin (TG). The intracellular calcium concentration is indicated by the color in the images. The blue/green in the scale bar indicates low levels of [Ca^2+^]I, and the yellow/red represents high [Ca^2+^]i. D) The group-averaged signals (mean ±*S.E.*) of the first seven calcium transients during the recording period for isolated cardiac cells.*, *P*<0.01 with respect to the caffeine group.

We investigated whether the SK2 channel and RyRs functionally interact in cardiac myocytes. The whole-cell I*_K,Ca_* current (apamin-sensitive current) was recorded from a single atrial myocyte from 9-11 animals for each group in the presence or absence of apamin (Sigma, Fig. 1A). The current density-voltage relations are summarized in Fig. 1B, which shows a significant decrease in the current density in the presence of a RyR2 inhibitor with 20 µM ryanodine (Alexis, n = 8 cells). The combination of the SR Ca^2+^ ATPase inhibitor thapsigargin (depletion of Ca^2+^ stores, 2 µM, Alexis) and ryanodine (20 µM, n = 8 cells) resulted in a significant negation of a component of the inward and outward currents in the atrial myocytes compared with the control cells (n = 10 cells, Fig. 1B). In another set of experiments, the application of caffeine (a gift from Dr. Zhang, the First Affiliate Hospital, Zhengzhou University, China) at 5 mM for 1 min prior to the electrophysiological recordings, which increased the Ca^2+^-sensitivity of RyRs and induced Ca^2+^ release from SR, caused a strong increase in the apamin-sensitive current density in the atrial myocytes (n = 6 cells, Fig. 1B).

To verify that these effects were associated with the RyR-sensitive Ca^2+^ release from SR stores, we performed a calcium-imaging experiment. Figure 1C shows representative samples of the line-scan images taken from a single atrial myocyte from 11 hearts elicited by caffeine under field stimulation. As shown in Fig. 1D, in the majority of myocytes, the fluorescence intensity of the [Ca^2+^]i elicited by caffeine was significantly suppressed by 20 µM of ryanodine alone (n = 9 cells) or by the combination of 20 µM ryanodine and 2 µM thapsigargin (n = 9 cells). There were significant differences in the normalized amplitude of the Ca^2+^ signal among the ryanodine alone, ryanodine and thapsigargin and control groups (p<0.01). There was no significant difference between the ryanodine group and the ryanodine and thapsigargin group (p>0.05, unpaired Student's *t* tests, Fig. 1D). The combination of our electrophysiological and calcium imaging data indicate that the application of ryanodine in this manner can inhibit intracellular calcium transients and associated SK channel currents, suggesting that ryanodine receptor-mediated Ca^2+^ release allows a sufficient increase in intracellular Ca^2+^ to increase the activation of SK2 channels in cardiac myocytes.

### Specific Involvement of SK2 in the knockdown of RyR2 mRNA

**Figure 2:**
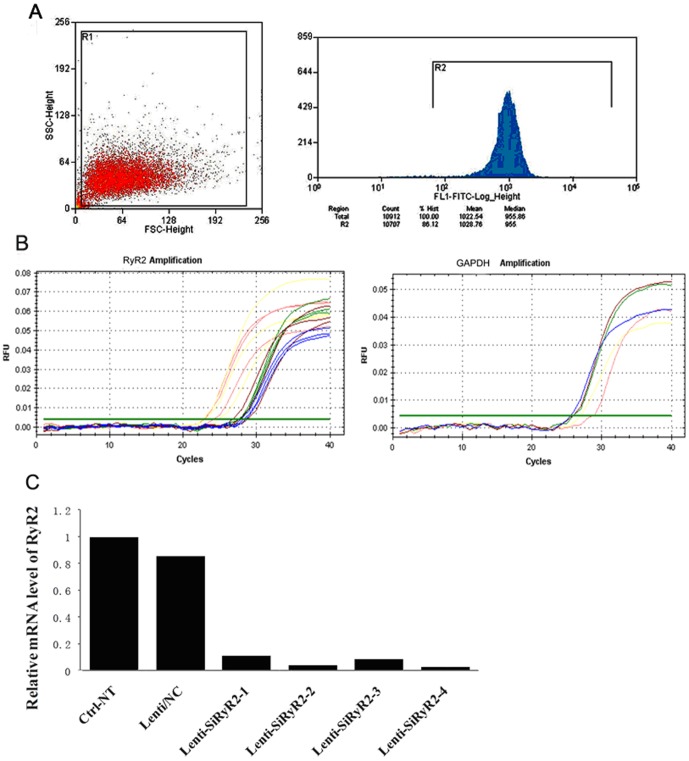
Downregulation of RyR2 in neonatal mouse cardiomyocytes by lentiviral-mediated siRNA specific to RyR2. A) Apoptosis and necrosis of the neonatal cardiomyocytes using flow cytometry analysis. The cells were treated for the indicated periods before annexin V and 7-AAD double staining. Q1, annexin V-negative/7-AAD-positive; Q2, annexin V-positive/7-AAD-positive; Q3, annexin V-negative/7-AAD-negative; Q4, annexin V-positive/7-AAD-negative. B) Amplification curves of RyR2 and GAPDH genes determined by real-time RT-PCR. C) The mRNA level of RyR2 was analyzed by real-time RT-PCR in neonatal cardiomyocytes treated with the recombinant Lenti-siRyR2 vectors (Lenti-siRyR2-1-4), scrambled siRNA (Lenti-NC) for 48 h and non-transfected cells (Ctrl-NT). There was a significant reduction in the RyR2 mRNA levels by lentiviral-mediated siRNA against RyR2 in neonatal mouse cardiomyocytes compared with the scrambled siRNA and non-transfected cells according to the real-time RT-PCR.

To further document the functional regulation of RyR2 on the SK2 channels in cardiac myocytes, we examined the effect of lentiviral-mediated siRNA RyR2-silencing on the SK2 channel in NMCMs. The total mRNA preparations of four different recombinants of lentiviral-mediated RyR2 knockdown were subjected to real-time PCR to examine the RyR2 mRNA levels. The infection efficiency of the neonatal mouse cardiac myocytes transduced with the lentiviral-mediated siRNA vectors was determined using GFP as the reporter gene. Greater than 91% of the cardiomyocytes were GFP positive based on flow cytometry (Fig. 2A). As shown in Fig. 2C, the lentiviral-mediated inhibition of RyR2 decreased the level of RyR2 mRNA in NMCMs, and the scrambled sequence of negative siRNA or the non-transfected cells had an effect on RyR2 expression in cultured neonatal myocytes based on real time-PCR analysis. The effective recombinant lentivirus products are defined as Lenti-siRNA-RyR2-1, Lenti-siRNA-RyR2-2, Lenti-siRyR2-3 and Lenti-siRyR2-4, respectively, and were used for further functional experiments.

**Figure 3:**
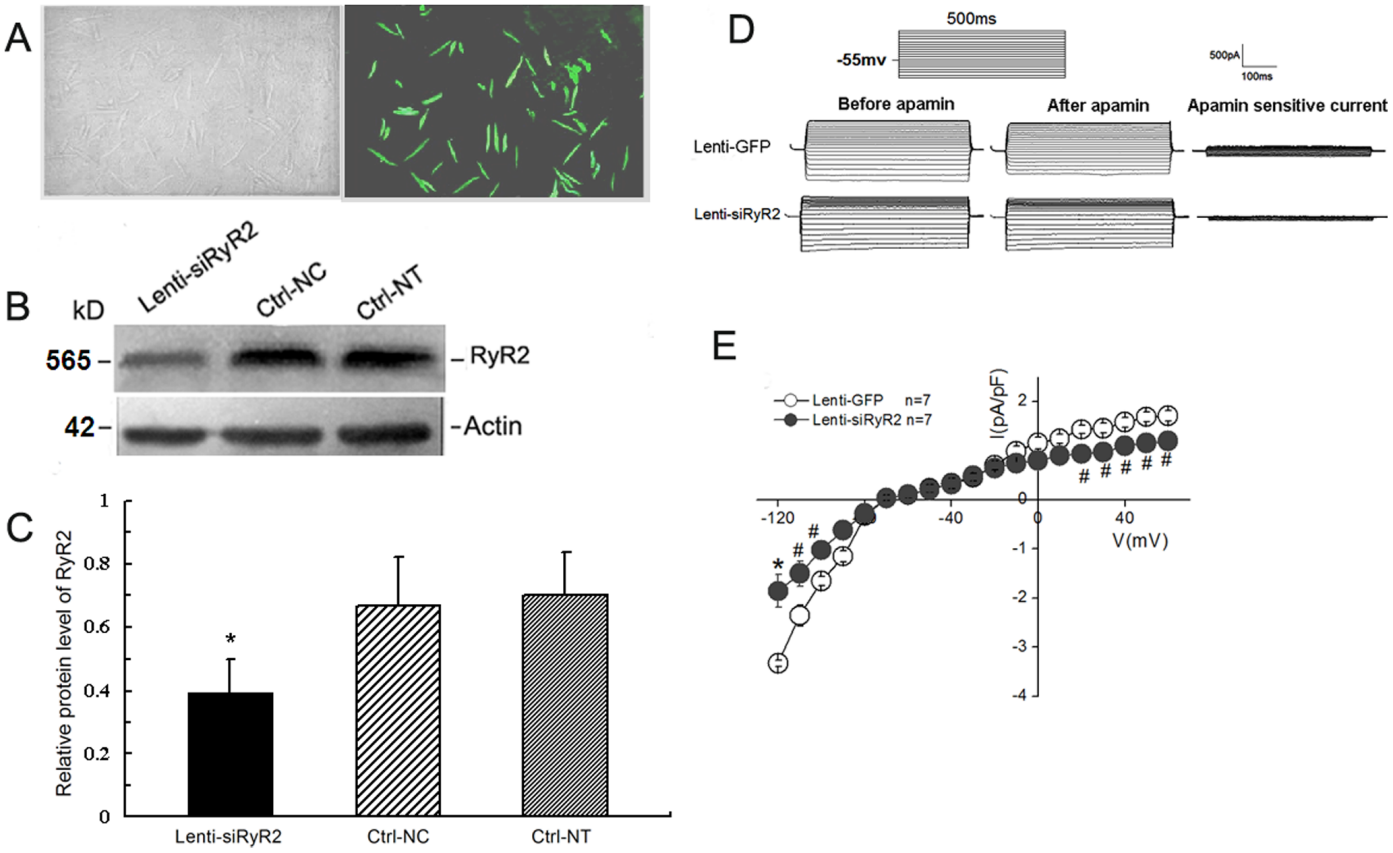
Knockdown of RyR2 on *I_K,Ca_* in mouse atrial myocytes transduced with recombinant lentivirus containing RyR2-siRNA. A) Photomicrographs showing the GFP expression in adult cardiomyocytes infected with the recombinant Lenti-siRNA-RyR2 vectors after 36 h under a fluorescent microscope. The corresponding contrast image is shown in the left panel. B) Western blot of RyR2 expression detected by monoclonal mouse anti-RyR2 antibody in adult cardiac myocytes infected with Lenti-siRyR2 vectors for 48 h. Ctrl-NC and Ctrl-NT represent the cells transfected with a scramble siRNA probe and the non-transfected cells, respectively. The 565-kDa bands represent RyR2. C) Quantification of the proteins was performed by densitometry, showing RyR2 knockdown in the cells. *,p<0.05 with respect to Ctrl-NT and Ctrl-NC groups, N = 5 for each. D) Samples of whole-cell *I_K,Ca_* recorded from mouse atrial myocytes transduced with recombinant Lenti-siRyR2 vectors (Lenti-siRyR2-2 and Lenti-siRyR2-4) using voltage steps from a holding potential of −55 mV. E) Summary data of the current density-voltage relations showing inhibition of the apamin-sensitive current by knockdown of RyR2.*,*P*<0.01, #,*P*<0.05 with respect to the control group.

We determined the effect of RNAi gene silencing on the expression of RyR2 in cultured adult cardiac myocytes using western blot analysis. Figure 3A shows the GFP expression in cardiac myocytes co-transfected with Lenti-siRyR2-2 and Lenti-siRyR2-4 for 48 h under a fluorescent microscope. The proteins from cultured cell preparations were analyzed using specific antibodies against RyR2, and the 565 kDa bands represent RyR2 (Fig. 3B). The RyR2 expression in the cardiac cells treated with siRNA vectors decreased by 41.31% and 44.10% of the levels observed in the cardiac myocytes infected with control siRNA vectors (Ctrl-NC and Ctrl-NT, respectively) (p<0.05, n = 5 for each, *ANOVA*, Fig. 3C).

**Figure 4:**
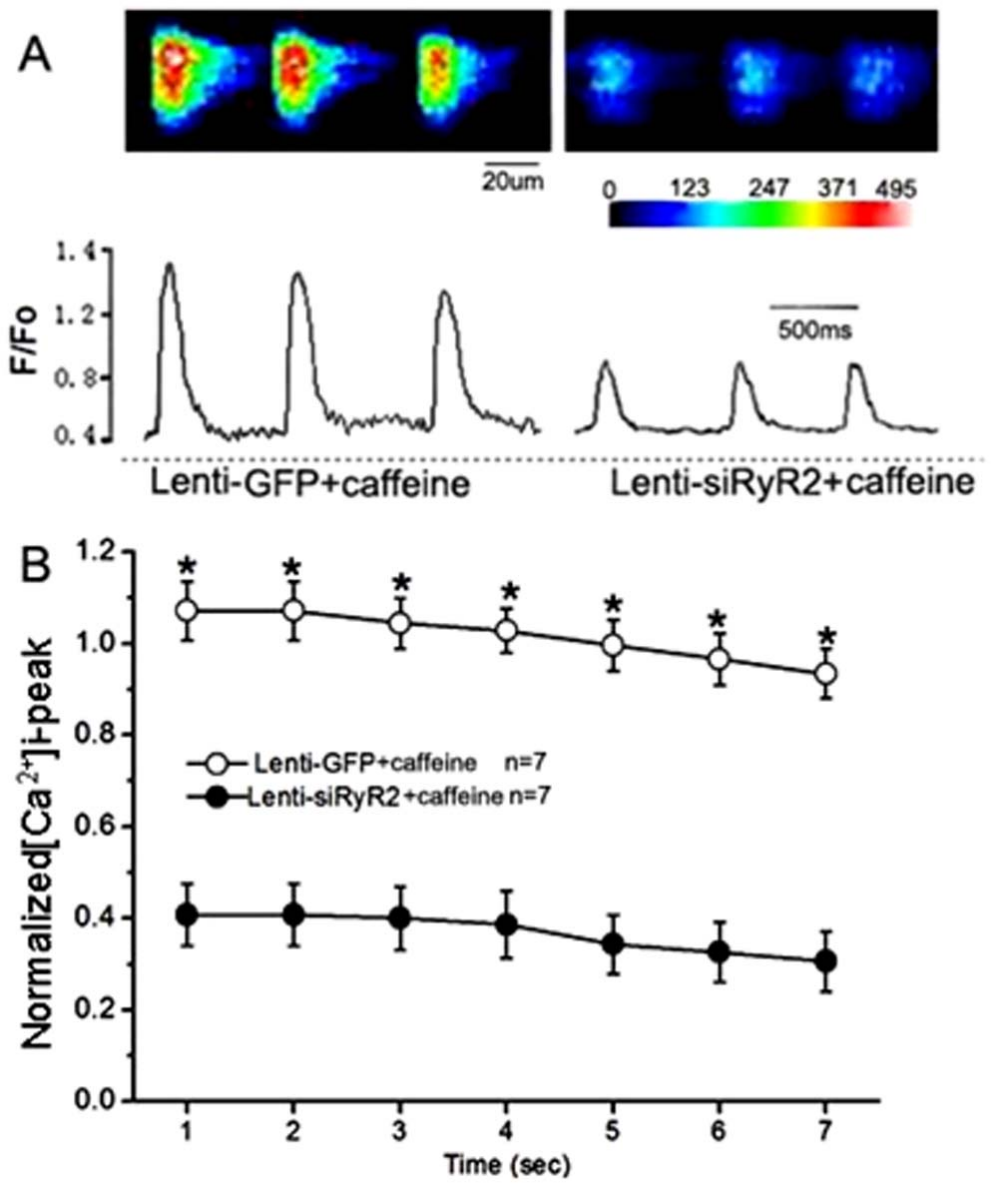
Effect of knockdown of RyR2 on the intracellular calcium transient (F/F_0_) in the atrial myocytes transduced with lentiviral-mediated siRNA specific RyR2. A) Examples of the line-scan images for the single atrial myocytes (top) and corresponding fluorescence intensity-time curves (below). The [Ca^2+^]i transient induced by 5 mM caffeine was suppressed by the knockdown of RyR2 in the atrial cells. The intracellular calcium concentration is indicated by the color in the images. The blue/green in the scale bar indicates low levels of [Ca^2+^]I, and yellow/red represents high [Ca^2+^]i. B) Group-averaged signals (mean ±*S.E.*) of the first seven calcium transients during the recording periods for isolated cardiac cells with different treatments.*, *P*<0.01 with respect to the caffeine group.

To directly examine the functional role of RyR2 knockdown on cardiac myocytes, whole cell patch-clamp techniques were used to record the I*_K,Ca_* in adult atrial cells co-transduced with Lenti-siRyR2 for 36–48 h. Figure 3D shows examples of the I*_K,Ca_* recorded from atrial cells transduced with Lenti-siRyR2 vectors. The current density-voltage relations are summarized in Fig. 3E. Compared with the control cells (n = 7 cells), the atrial myocytes transduced with Lenti-siRyR2 vectors (n = 7 cells) revealed a significant decrease in the apamin-sensitive current density (p<0.05, p<0.01 for the different depolarization potentials, unpaired Student's *t* tests, Fig. 3E). We tested whether the knockdown of RyR2 affected the [Ca^2+^]i fluorescence intensity using confocal imaging. As shown in Fig. 4A, there was a decrease in the [Ca^2+^]i fluorescence intensity in the cardiac myocytes transduced with a lentiviral-mediated siRNA specific to RyR2. The summarized data are displayed in Fig. 4B, illustrating that the knockdown of RyR2 significantly decreased the peak of the [Ca^2+^]i transient amplitude during the first seven calcium transients during the recording periods compared with the control group (n = 7 cells for each, p<0.01, unpaired Student's *t* tests). Our data further suggest a functional role of RyR-mediated Ca^2+^ release evoked by the Ca^2+^ influx on the SK channel activation in the cardiac myocytes.

### SK2 Associates with RyR2 in Native Tissue

**Figure 5:**
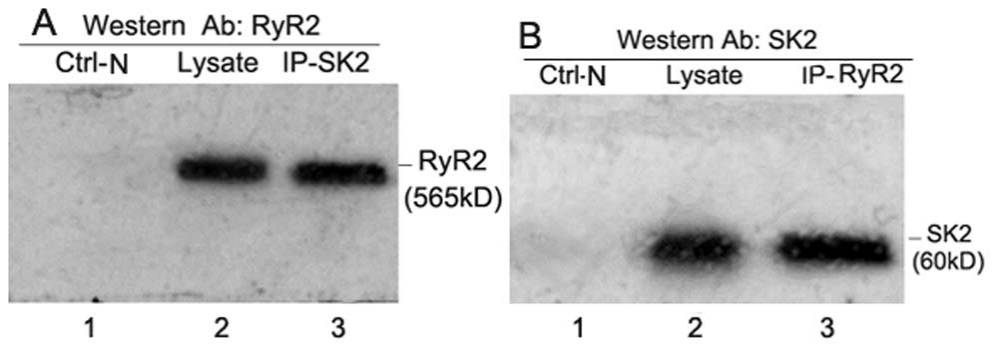
SK2 interacts with RyR2 *in vivo* in mouse atrial tissue. *A*–*B*) *in vivo* co-immunoprecipitation (IP) assays in the mouse atria. The specific and solubilized total proteins from the mouse atria were precipitated with anti-SK2 or anti-RyR2 antibodies. A) For RyR2, the immunoprecipitated complex was analyzed by 5% SDS-PAGE and identified by immunoblotting (IB) with anti-RyR2 antibodies (MW≈565 kDa, lane 3). B) For the SK2 protein, the immunoprecipitated complex was analyzed by 10% SDS-PAGE followed by IB with anti-SK2 antibodies. The SK2 proteins from the mouse atria (MW approximately 60 kDa) are in lane 3. Lane 2 is the immunoblotting result obtained using atrial lysate, confirming the presence of the SK2 protein (B) or the presence of the RyR2 protein (A) as positive controls. Lanes 1 (Ctrl-N, Fig. 5A and Fig. 5B) indicates the negative control using a non-immobilized gel.

The above results suggest the functional interaction of the SK2 channel with RyR2, but little is known regarding whether RyR2 associates with RyR2 in native mouse cardiac tissues. We examined the association of endogenous SK2 and RyR2 proteins in adult mouse cardiac muscle using an *in vivo* co-immunoprecipitation assay. The cell lysate from adult mice atria was isolated, immunoprecipitated with anti-SK2 antibodies, and immunoblotted with anti-RyR2 antibodies. As shown in Fig. 5A, RyR2 was selectively detected in the mouse atria, and no signal was detected in the negative control immunoprecipitates using a non-immobilized gel (Ctrl-N). Under these conditions, we performed similar immunoprecipitation with anti-RyR2 antibodies, followed by immunoblotting with anti-SK2 antibodies. The results displayed in Fig. 5B show that the SK2 protein, which had an expected size of 60 kDa, was identified in the mouse atria tissue, and there was no significant signal in the control immunoprecipitates. Co-immunoprecipitation of SK2 with RyR2 was achieved in the mouse cardiac muscle. We propose the presence of a SK2 and RyR2 complex in native cardiac tissues, even though SK2 did not directly bind to RyR2 in a yeast two-hybrid assay (data not shown). These results contribute to the evidence supporting functional communication between the SK2 channels and RyR2 Ca^2+^ release. The structural platform for the channels' crosstalk might be the association with junctional membrane complexes [20].

## Discussion

We used isolated mouse atrial myocytes and cultured cardiac myocytes infected with lentiviral-mediated small interference RNA targeting RyR2 to investigate the functional communication between the SK2 channels and RyRs-sensitive Ca^2+^ release. Our results show that the inhibition of the RyR2 Ca^2+^ release channel and the inhibition of the SERCA suppressed the SK2 channel current in cardiac myocytes. Activation of RyRs by caffeine increased the apamin-sensitive currents in the cardiac cells. This report illustrates the role of the RyRs-induced Ca^2+^ release from the sarcoplasmic reticulum in SK2 channel activation in cardiomyocytes.

In the present study, we used electrophysiological techniques to determine that the SK2 channel current is a time-independent inward rectifier of K^+^ current, which is consistent with previous reports [4], [7], [17]. In a variety of cells, the amplitude and time course of SK channel currents depends on the dynamics of [Ca^2+^]i and on the subcellular location of the SK channels [9]. Dr. Chiamvimonvat and coworkers observed that the cells were held at −55 mV and stepped to +10 mV for 30 ms to activate a L-type Ca^2+^ channel-mediated Ca^2+^ current and initiate the release of Ca^2+^ from the sarcoplasmic reticulum [17]. The cells were then stepped from +10 mV to +60 mV to increase the driving force for K^+^ while decreasing the driving force for the Ca^2+^ current. A component of the mainly outward current reflected the changes in the intracellular Ca^2+^ concentration in the cardiac myocytes [4]. Consistent with their observations, our electrophysiological data (Fig. 1B) showed significant differences in the outward component of the SK currents in the presence of caffeine, ryanodine, and thapsigargin. An earlier study demonstrated the effects of different [Ca^2+^]i concentrations on the Ca^2+^-activated potassium current [4]. One thousand nanomolar [Ca^2+^]i significantly increased the Ca^2+^-activated potassium current in single cardiac cells compared to the concentration of 100 nm. In the present study, caffeine increased the Ca^2+^-sensitivity of RyRs and Ca^2+^ release from SR by elevating the amplitude of the Ca^2+^-activated potassium current. The inhibition of RyR2 by blocking it or by knockdown of RyR2 mRNA suppressed the Ca^2+^-activated potassium current. These findings suggest that blocking RyR2 might lead to a diminished Ca^2+^ supply for SK channel activation. The dynamics of [Ca^2+^]i could result in a change in the sensitivity of SK channels to transient Ca^2+^ release events [9], [21], [22]. This might affect the open probability and the opening state of the SK channels.

A previous study demonstrated that the SK2 channels in cardiac muscle are mainly coupled to the Cav1.3 channel [17]. Ca^2+^ entry through the voltage-gated Ca^2+^ channels activates the SK channels in cardiac cells. Ca^2+^ influx through the VDCCs stimulates the opening of RyR2 and subsequent Ca^2+^ release from the SR [23]. The method of functional communication between the SK channels and SR Ca^2+^ that occurs in cardiac cells is not known.

We summarize the three principal findings that support our conclusions. First, we demonstrated the functional modulation of the RyR2 channel on the Ca^2+^-activated potassium current in the mouse atrial cells by a RyR2 blocker and a RyR2 activator. We found that the apamin-sensitive current in the mouse atrial cells was abolished by siRNA RyR2 knockdown. Second, we found that the intracellular Ca^2+^ transient contributed to our understanding of the dependence of SK channel activity on SR Ca^2+^ release. Third, we tested the interaction between SK2 and RyR2 in mouse cardiac muscle using co-immunoprecipitation. RyR2 did not interact directly with the SK2 channel, as suggested by the YTH screen, but both proteins were present in native cardiac tissue as members of the same immunoprecipitated complex. All these phenomena lead to the conclusion that ryanodine receptor-mediated Ca^2+^ release activates small-conductance Ca^2+^-dependent K^+^ channels in cardiac myocytes. The combination of the published report [17] and our data suggests that the SK2 channels in cardiac myocytes could be activated by a Ca^2+^ influx directly through voltage-gated Ca^2+^ channels or indirectly through ryanodine receptor-mediated Ca^2+^ release from SR intracellular stores.

It was reported that the SK2 channels profile the cardiac action potential repolarization, especially during the late phase of the cardiac action potential [4], [6]. In cardiomyocytes, the effective distance between Cav1.2 and RyRs is estimated to be <100 nm [24], and the CICR can be triggered in a few milliseconds [25], [26]. This property can result in fast coupling of RyRs Ca^2+^ release to the SK channels in the cardiac myocytes. Giant inside-out patches showed that the onset of the SK current commences within 1 ms after Ca^2+^ application, with time constants of the SK channels activation of 5–15 ms and the time constants of deactivation of 22–38 ms [11]. Because the SK channels are gated by intracellular Ca^2+^ ions only, the SK current could be repeatedly activated for as long as the patches remained intact without changes in the activation kinetics [11]. In our experiment, the apamin-sensitive current represents a certain amount of the total sustained current (Fig. 1A), but we predict that the SK-mediated Ca^2+^-activated K^+^ current remains active after a transient outward K^+^ current (*I*to) is inactivated [4]. The ablation of the SK2 channel results in a significant prolongation of the terminal portion of the repolarization phase in the cardiac myocyte and atrial fibrillation [6]–[8]. The SK2 channel -mediated current was responsible for the late phase of the action potential.

SK channels have been shown to mediate after hyperpolarizations [27], [28]. RyR2, the predominant RyR isoform in cardiac muscle, is an essential protein for the excitation-contraction (EC) coupling in cardiac muscle through its mediation of Ca^2+^ release and altered intracellular Ca^2+^ load. During the initiation of contraction, the modulation of RyR2 on the SK2 channel in cardiac myocytes contributes significantly to the cardiac electrical-mechanical cycle. It is important to define the functional communication between the SK2 channel and RyR2 in the cardiac myocytes because this communication indicates a new signaling pathway for SK channels by RyRs-induced Ca^2+^ release and signaling in the heart.

## Supporting Information

Text S1 Supplementary Methods.
(DOC)
