# Supplementary material for: RyR2 Modulates a Ca2+-Activated K+ Current in Mouse Cardiac Myocytes
Source: PLoS One. 2014 Apr 18;9(4):e94905. doi: 10.1371/journal.pone.0094905 (PMC3991633; doi:10.1371/journal.pone.0094905)
Supplement: Text S1 — Supplementary Methods. (DOC) [file pone.0094905.s001.doc]

**Supplementary Methods**

**Single cardiac myocyte isolation**

Adult C57B L mice were anesthetized with sodium pentobarbital (80 mg/kg, intraperitoneally). The hearts were quickly removed from the chest and perfused via the Langendorff method with Ca^2+^-free modified Tyrode’s solution containing (mmol/L) NaCl 140, KCl 5.4, MgCl_2_ 1, HEPES (N-2-hydroxyethylpiperazine-N-2-ethanesulphonic acid) 10 and glucose 10 at pH 7.4 for 5 min until the blood was washed out. The hearts were perfused with a fresh enzyme solution with collagenase type II (Sigma-Aldrich) and protease (type XIV, Sigma-Aldrich). After 30-40 min, the solution was changed to a high-K^+^ solution (mmol/L: potassium glutamate 120, KCl 20, MgCl_2_ 1, EGTA 0.3, glucose 10 and HEPES 10, pH 7.4 with KOH) for 5 min. The entire perfusion procedure was performed at 37℃，100% O_2_. After perfusion, the heart was removed from the Langendorff apparatus and the atria and ventricles were separated. Single cells from the atria were kept in the high-K^+^ solution for 2 h at room temperature before electrophysiological recordings.

**Electrophysiological recordings**

The whole-cell configuration of the patch-clamp technique was used as previously described [1]. The Ca^2+^-activated K+ current (*I* _K_*,*_Ca_) was recorded from freshly isolated atrial myocytes at room temperature using a voltage-clamp protocol as previously described [1]. The bath solution contained (mmol/L) *N*-methylglucamine (Sigma), 140; KCl, 4; MgCl_2_, 1; glucose, 5; and Hepes, 10 (pH 7.4 using methane sulfonic acid). The whole-cell configuration involved the use of a glass pipette with a tip resistance of 4–5 MΩ filled with an internal pipette solution of (mmol/L) potassium gluconate, 144; MgCl_2_, 1.15; EGTA, 5; HEPES, 10. Five hundred nanomoles of free intracellular calcium was applied by a calcium titration program used to calculate the free, bound and dissociated [Ca^2+^]. The pH was adjusted to 7.25 with KOH. An EPC-10 (HEKA Elektronik, Germany) patch-clamp amplifier was used with Pulse 8.67 software (HEKA Elektronik, Germany). In all the experiments, a series resistance compensation of ≥90% was obtained. The currents were normalized to the cell capacity to obtain the current density (pA/pF).

**Confocal images**

The calcium-imaging experiments were performed as described elsewhere [2]. The [Ca^2+^]i transients were obtained either from freshly isolated single cardiomyocytes or from cultured myocytes transduced with Lenti-siRyR2 vectors previously loaded with the fluorescent Ca^2+^ dye Fluo-3AM (15 μmol/L, Molecular Probes) for 20 min at room temperature. The experiments were divided into five groups, including the control (caffeine alone), ryanodine+caffeine, ryanodine+thapsigargin+caffeine, Lenti-GFP+caffeine (control) and Lenti-siRyR2+caffeine. The control cells were dispersed in Tyrode’s solution (mmol/L: 140 NaCl, 4 KCl, 1.1 MgCl_2_, 10 Hepes, 10 glucose, 1.8 CaCl_2_; pH 7.4 with NaOH), and the other groups were dispersed in Tyrode’s solution supplemented with different individual inhibitors before the experiment. All the cells were stimulated at 1 Hz until reaching steady state, and 5 mM caffeine was then applied to evoke the [Ca^2+^]i transients. The image data were recorded over 1-2 min following the caffeine application. All the imaging data were taken in the line-scanning mode along the long axis of the myocyte excited at 488 nm with a confocal laser scanning microscope (Olympus FV1000, Japan) and analyzed by Olympus Fert (Japan). The Ca^2+^ level was reported as fluorescence (*F*) over fluorescence min (*F*_o_), where F_0_ is the resting or diastolic fluo-3 fluorescence.

**Cell Culture**

293T cells were cultured in Dulbecco’s modified Eagle’s medium (DMEM，GIBCO) supplemented with 10% fetal bovine serum (FBS, Invitrogen) and 1% penicillin-streptomycin (PS, Invitrogen), and they were maintained at 37°C in a humidified atmosphere containing 5% CO_2_. The cells were subcultured at 1 × 10^5^ cells per well into six-well tissue culture plates. After 24 h of culture, the cells were infected with recombinant lentiviral vectors.

**Construction of small interfering RNA**

To knockdown the RyR2 mRNA in cardiac myocytes, four optimal 19-mer target sequences (GCCATTCCTACAGTGGTAT, location 864, CGTCCACATACTATTACTC, location 4767, AGGACACCATCAATCTGCT, location 6837 and CACAGCCTATCATCAACAA, location 10404) were selected based on the cDNA sequence of mouse RyR2 (accession number NM-023868.2). BLAST searches confirmed that the selected oligonucleotide sequences did not possess homology to any other genes. Four pairs of oligonucleotides encoding shRNAs and a negative control shRNA were designed and chemically synthesized by Invitrogen based on the targeting sequences. These sequences were subcloned into the HIV-based *psi*HIV-U6 plasmid vector (Guangzhou GeneCopoeia, China). The plasmid contained a reporter gene, GFP, under the control of a separate CMV promoter (Guangzhou GeneCopoeia, China). The recombinants were identified by electrophoresis and sequencing.

Five recombinant lentiviral vectors were packaged and amplified in 293T cells using the Lenti-Pac^TM^ HIV Expression Packaging Kit (Clontech) according to the manufacturer’s protocol to generate Lenti-siRNA-RyR2-1, Lenti-siRNA-RyR2-2, Lenti-siRNA-RyR2-3, Lenti-siRNA-RyR2-4, Lenti-siRNA-NA (negative control). The culture supernatant containing lentiviral particles was collected following transfection for 48 h. The supernatant was centrifuged at 500 rpm for 10 min to remove cell debris and filtered with a 0.45 μm polyethersulfone funnel with low protein adherence. The resulting pellet was resuspended in PBS, and the lentiviral stocks (> 10^7^ units/ml) were stored at -80℃ until use.

**Mouse neonatal cardiomyocyte culture and infection**

Primary cultures were prepared from newborn mouse hearts using a technique modified from Simpson et al [3]. Twenty-four hour old neonatal mice hearts were dissected from the chest. The blood vessels and atria were removed, and the remaining ventricular tissues were minced into approximately 1 mm^3^ pieces and washed in a Ca^2+^- and Mg^2+^-free Hanks’ balanced salt solution (GIBCO). The tissue pieces were subjected to sequential digests with an enzyme solution of 1 mg/ml trypsin and 50 U/ml DNase II (Sigma) and stirred continuously at 37°C. Following the trypsin digestion, the supernatant from the digests was collected into conical polycarbonate tubes containing 1:1 FBS every 3-5 min until the ventricular tissues were relatively dissolved. Once the dispersed cells were pelleted, the cells were resuspended and pre-plated onto 100-mm dishes in DMEM with 10% FBS for 2 h. The unattached myocytes were plated in 35-mm dishes in MEM with 10% FBS, 1% PS, 0.1 mmol/L bromodeoxyuridine (BrdU, Sigma), and 20 μmol/L arabinosylcytosine (Ara-C, Sigma) at 37°C in a 5% CO_2_ incubator. After 24 h, the culture media was changed. The cells were maintained at 37°C in a 5% CO_2_ incubator.

Following the fusion of 50% of the cultured neonatal mice cardiac myocytes (NMCMs), the cells were collected by trypsin digestion and cultured in serum-free MEM. The recombinant shRNA lentiviral products were added to each culture dish, and the cells were maintained at 37°C in a 5% CO_2_ incubator for 48 h. The infecting efficiency of the NMCMs was detected by flow cytometry.

**Flow Cytometry**

NMCMs infected with shRNA lentiviral vectors were collected after digestion with trypsin and washed with cold PBS. The cells were resuspended in 200 μL of PBS in a culture tube. The infected cells expressing both the siRNA and GFP were monitored by GFP fluorescence using an FCM machine (BD FACSCanto II, USA).

To identify apoptotic and dead cells in the NMCMs infected with the lentiviral vectors, a PE Annexin V Apoptosis Detection Kit I (BD Pharmingen™) was used according to the manufacturer’s directions. The cells were suspended in 100 μl of 1X Annexin V binding buffer at a concentration of 2 x 10^5^ cells/ml in a 5 ml culture tube. Five microliters of PE Annexin V and 5 μl of 7-amino-actinomycin D (7-AAD) were added to each sample. After a 15-min incubation at room temperature in the dark, 400 μl of 1X Annexin V binding buffer was added to each tube. The flow cytometry analysis was performed using a FACS Calibur system with FACSDiva software (BD FACSCanto II, USA).

**Real-time quantitative PCR**

Total RNA from the neonatal myocytes infected with the recombinant shRNA lentivirus vectors was extracted using the Trizol Reagent (Invitrogen). The isolated RNA was treated with DNase at 37°C for 30 min to remove any contaminating genomic DNA, if present. cDNA was synthesized from the total RNA samples by oligo (dT)-primed reverse transcription using the RevertAid^TM^ First Strand cDNA Synthesis kit (Fermantas). The quantitative real-time polymerase chain reaction (PCR) products were detected using a SYBR Green qPCR Kit (TAKARA) by Bio-Rad MiniOption (USA). The PCR primers used to detect RyR2 and GAPDH were: RyR2 (251 bp), Forward: 5'-GAATTCATCATGGATACTCTACC-3' Reverse: 5'-GTCATGCACATTATCTTCTGCAT-3'; GAPDH (150 bp), Forward: 5'-TGTGTCCGTCGTGGATCTGA-3', Reverse: 5'-TTGCTGTTGAAGTCGCAGGAG-3'. All RyR2 and GAPDH primers were desired and synthesized by Shanghai Bioasia Company (China). The relative quantification of RyR2 mRNA was analyzed using the formula: Fold=2^-△△Ct^, △Ct= Ct(target)–Ct(ref), where ref: GAPDH, target: gene of interest, and Ct: threshold cycle. The expression of RyR2 was determined by the normalization of the threshold cycle (C_t_) of these genes to the control housekeeping gene.

**Lentiviral transduction of adult mouse cardiac myocytes**

Freshly isolated atrial myocytes were suspended in modified Tyrode’s solution containing (mmol/L): 113 NaCl, 4.7 KCl, 0.6 KH_2_PO_4_, 0.6 Na_2_HPO_4_, 1.2 MgSO_4_, 12 NaHCO_3_, 10 KHCO_3_, 10 HEPES, 30 taurine, 5% FBS and 12.5 μM Ca^2+^ for 10 min at room temperature. After the myocytes were pelleted by gravity, the myocytes were resuspended in the above solution, and the Ca^2+^ concentration in the myocyte suspension was slowly increased to 1 mM.

Culture dishes were precoated with 10 mg/ml mouse laminin (GIBCO) in phosphate-buffered saline with 1% PS at room temperature for 1 h. The isolated cardiac myocytes were suspended in MEM containing 1.2 mM Ca^2+^, 2.5% FBS, and 1% PS (pH 7.35–7.45). After the myocytes were pelleted by gravity for 10 min, the supernatant was aspirated, and the myocytes were washed two times using the same protocol. The myocytes were plated in a 60-mm laminin-coated dish in MEM containing 2.5% FBS and 1% PS. After 1 h of culture in a 5% CO_2_ incubator at 37℃, the medium was changed to FBS-free MEM, and an appropriate titer of gene-carrying lentivirus was added to the medium. The cells were maintained at 37°C in a 2% CO2 incubator for 36-48 h. A recombinant lentivirus containing GFP alone was used as a control for our experiments. The GFP-positive cells were used for the electrophysiological recordings.

**Western blotting**

Cultured adult mouse cardiac myocytes were harvested 48 h after the transduction of the recombinant lentivirus vectors and homogenized in ice-cold modified lysis buffer (mmol/L) containing Tris-HCl (pH 7.4) 50, NaF 20, Na_3_VO_4_ 1, NaCl 200 and 0.5% Triton X-100 and protease inhibitors (Roche). The supernatant was collected by centrifugation at 1,000 x g for 5 min at 4°C to remove cell debris. Thirty-five microgram aliquots (measured using a BCA protein assay regent kit, Pierce) from the protein samples were separated by 5% gradient SDS-PAGE and transferred to polyvinylidene difluoride (PVDF) membranes (Bio-Rad). Following transfer, the membrane was blocked in 5% non-fat dried milk (wt/vol) in Tris-buffered saline with 0.1% Tween-20 (TBS-T) at room temperature for 2 h. After blocking, the membranes were incubated with anti-RyR2 antibody (diluted 1:600, Affinity BioReagents) at 4℃ overnight. The membrane was incubated with horseradish peroxidase-conjugated anti-mouse IgG secondary antibody (Thermo). The bands were detected by chemiluminescence (Thermo). Quantification of the signals was performed by densitometry (The Discovery Series™ Quantity One® 1-D Analysis Software Version 4.6.2).

**Co-immunoprecipitation**

Reciprocal Co-IP was performed as previously described [4]. The protein samples were extracted from adult mouse atrial tissues. Three hundred microliters of soluble protein was incubated with anti-SK2 antibody or anti-RyR2 antibody overnight at 4℃ followed by an additional incubation with 30 μl protein G sepharose (Santa Cruz) for 6 h at 4°C. The immunoprecipitated complexes were obtained by centrifugation and washed four times with a high stringency buffer (0.1% SDS, 1% deoxycholic acid, 0.5% Triton X-100, 20 mM Tris-HCl, 120 mM NaCl, 25 mM KCl, 5 mM EDTA, 5 mM EGTA, 0.1 mM DTT with 17.1 g sucrose in 200 ml), then with a high-salt buffer (2.922 g NaCl in 200 ml high stringency buffer) and then with a low-salt buffer (2 mM EDTA, 0.5 mM DTT, 10 mM Tris-HCl) to eliminate non-specific binding. The precipitated proteins were analyzed with western blotting using SK2 or RyR2 antibodies.

Western blots were performed as previously described in the methods section. Each sample containing equal amounts of proteins (35 μg/ lane) underwent 10% or 5% SDS-PAGE (SK2 and RyR2, respectively) and were transferred to PVDF membranes. Following transfer, the membrane was blocked and incubated with anti-SK2 (diluted 1:200, Sigma-Aldrich) or anti-RyR2 antibodies (diluted 1:600) at 4℃ overnight. The membrane was hybridized with horseradish peroxidase-conjugated anti-rabbit IgG or anti-mouse IgG secondary antibody (Thermo), which was detected by chemiluminescence (Thermo).

**References**

1. Zhang Q, Timofeyev V, Lu L, Singapuri A, Long MK, et al. (2008) Functional roles of a Ca^2+^-activated K^+^ channels in atrioventricular node. Circ Res 102: 465–471.
2. Pereira L, Metrich M, Fernandez-Velasco M, Lucas A, Leroy J, et al. (2007) The cAMP binding protein Epac modulates Ca^2+^ sparks by a Ca2+/calmodulin kinase signalling pathway in rat cardiac myocytes. J Physiol 583: 685–694.
3. Simpson P and Savion S. (1982) Differentiation of rat myocytes in single cell cultures with and without proliferating nonmyocardial cells. Cross-striations, ultrastructure, and chronotropic response to isoproterenol. Circ Res 50: 101-116.
4. Lu L, Zhang Q, Timofeyev V, Zhang Z, Young JN, et al. (2007) Molecular coupling of a Ca^2+^-activated K^+^ channel to L-type Ca^2+^ channels via alpha-actinin2. Circ Res 100:112–120.
